# Mixing Safety of Composite Solid Propellant Slurry in a Blade-Free Planetary Mixer

**DOI:** 10.3390/ma19081672

**Published:** 2026-04-21

**Authors:** Yuncheng Li, Qingjun Wang, Hanyu Chen, Yuanwei Xi, Weibin Tao, Dayong Li, Min Xia

**Affiliations:** 1 School of Materials Science and Engineering, Beijing Institute of Technology, Beijing 100081, China; 3220231524@bit.edu.cn (Y.L.);; 2 Liaoning Qing Yang Chemical Industry Corporation, Liaoyang 111002, China; 3 China North Chemical Research Academy Group Co., Ltd., Beijing 100089, China; 4 Key Laboratory of High Energy Density Materials, Ministry of Education, Beijing Institute of Technology, Beijing 100081, China

**Keywords:** composite solid propellant, Blade-Free Planetary Mixer, mixing process, safety, mechanical sensitivity

## Abstract

Blade-Free Planetary Mixer (BFPM) can rapidly and efficiently mix highly viscous materials because of the strong centrifugal forces generated by the planetary motion of the mixing vessel. The safety of energetic propellant slurry during BFPM processing is critical. In this work, the mixing performance and process safety of composite solid propellant slurry in a BFPM were investigated through morphology observation, mixing index analysis, temperature measurement, rheological testing, mechanical sensitivity evaluation, and thermal analysis. The results showed that the BFPM achieved safe, efficient, and uniform mixing of the slurry. Under the baseline condition of 1000 rpm, the mixing index reached 95.73% after 24 min, and the slurry temperature increased to only 31.1 °C. The influence of BFPM processing on slurry safety was mainly reflected in the spatial redistribution of energetic solid components and the solid–liquid mixing state. And mechanical sensitivity tended to increase in regions of higher apparent viscosity. Increasing the rotational speed and adopting alternating rotation promoted particle dispersion and reduced local apparent viscosity, but an excessively high rotational speed reduced thermal stability. Overall, 1200 rpm combined with alternating rotation was identified as the most suitable operating condition. This work provides a practical basis for the safe and efficient BFPM processing of energetic propellant slurries.

## 1. Introduction

Solid propellants are the main energy source of solid rocket motors. They are composed of oxidizers, polymeric binders, plasticizers, and various functional additives [1,2,3]. The mixing uniformity of these components directly affects the combustion behavior and mechanical properties of the final propellant. Mixing is therefore a critical step in solid propellant processing. Nitrate ester plasticized polyether propellants have attracted widespread attention and have been increasingly used because of their high energy levels [4,5,6]. However, because they contain large amounts of energetic nitramine components, and conventional vertical mixing often involves strong blade-induced shear, friction against the vessel wall, and local energy accumulation, processing safety becomes a major concern.

To address this challenge, blade-free mixing technologies have emerged as promising alternatives to conventional blade-driven mixing systems [7,8,9,10,11]. Among them, the Blade-Free Planetary Mixer (BFPM) has attracted increasing attention in recent years as an efficient mixing technology. In BFPM, the vessel undergoes simultaneous rotation and revolution with an inclined axis configuration, which improves mixing performance. This coupled motion generates strong centrifugal forces and three-dimensional flow, allowing efficient mixing, deaeration, and homogenization without blades [8,9,12,13,14]. Owing to this motion mode, the BFPM offers distinct advantages in reducing blade wear, minimizing contamination and mechanical damage, and processing highly viscous systems. As a result, BFPMs have been widely applied in pharmaceuticals, food processing, and fine chemical engineering, and have shown particularly great potential for the mixing of highly viscous fluids and particulate systems [15,16,17].

Research on BFPMs initially focused on their feasibility and application potential [8,18,19,20]. Massing et al. demonstrated that dual asymmetric centrifugation could efficiently prepare liposomes in a closed container and showed that rotational speed, processing time, material concentration, and auxiliary mixing media all influenced the final product size and uniformity [9]. Subsequent studies also confirmed that planetary or centrifugal mixing can effectively improve dispersion quality and reduce gas entrapment in pharmaceutical powders and slurry-like materials [21,22]. Meanwhile, the understanding of BFPM mixing mechanisms has continued to advance. Chergui et al. used CFD to demonstrate that the flow inside a BFPM deviates from simple solid-body rotation and evolves into a complex three-dimensional vortical structure [13]. Yamagata et al. further demonstrated through particle dispersion measurements, planar PIV, and three-dimensional tracer tracking that an optimum precession rate exists in a planetary mixer [23]. Under these conditions, highly three-dimensional flow structures and chaotic advection are most pronounced, leading to the best mixing performance, whereas an excessively high precession rate may weaken mixing due to the growth of low-velocity regions. More recently, Son and Gao extended BFPM research to cohesive particles, viscous polymer resins, and free-surface flow [24,25,26]. Their results indicated that the rheology of the processed material strongly affects local flow patterns, coherent structures, free-surface evolution, and shear-rate distribution, and that high-shear regions often develop near the vessel wall.

In the field of solid propellants, researchers have performed mixing experiments using the BFPM. After mixing at 2000 rpm for 6 min, they obtained a propellant slurry with good homogeneity and a safe temperature [27]. Despite these advances, existing BFPM studies have mainly focused on mixing uniformity, flow structure, and application feasibility. Systematic studies on the safety of energetic propellant slurries during BFPM processing remain limited. For composite solid propellant slurries, process safety is not only related to temperature rise, but also to the dynamic redistribution of energetic solids, the wetting and coating state of particles, local apparent viscosity, mechanical sensitivity, and thermal stability [28,29,30]. These variables evolve simultaneously during mixing and may significantly influence one another. However, studies that evaluate BFPM safety by combining mixing state, rheological evolution, mechanical sensitivity, and thermal decomposition behavior remain scarce.

Based on the above considerations, this work investigates the safety of composite solid propellant slurry during BFPM processing. Mixing performance is first characterized to determine the slurry homogenization behavior. The changes in temperature, apparent viscosity, mechanical sensitivity, and thermal stability during the mixing process are then analyzed to clarify the dominant factors governing process safety. In addition, the effects of key operating parameters, including rotation speed and mode, are comparatively studied in order to identify effective routes for improving the safety of BFPM-based propellant mixing.

## 2. Materials and Methods

### 2.1. Materials

Hydroxyl-terminated poly (epichlorohydrin-co-tetrahydrofuran) random copolyether (PET, M_n = 4038 g/mol, average functionality = 1.76, hydroxyl value = 24.58 mg KOH/g, vacuum-dried at 60 °C for 48 h) and N-n-butyl-N-(2-nitroxyethyl) nitramine (Bu-NENA, 99%, M_n = 207.2 g/mol) were supplied by Liming Chemical Research Institute (Luoyang, China). Ammonium perchlorate (AP, Class III, mean particle size = 123 μm) and hexogen (RDX, mean particle size = 45 μm) were purchased from Liaoning Qing Yang Chemical Industry Corporation (Liaoyang, China). Aluminum powder (Class III, mean particle size = 5.3 μm) was purchased from Tianjin Aluminum Industry Co., Ltd. (Tianjin, China). All solid particles were dried at 60 °C for 7 d before use. The formulation used in the mixing process was kept constant, with a solid loading of 73.5 wt%.

### 2.2. Methods

#### 2.2.1. Mixing Conditions

**Figure 1:**
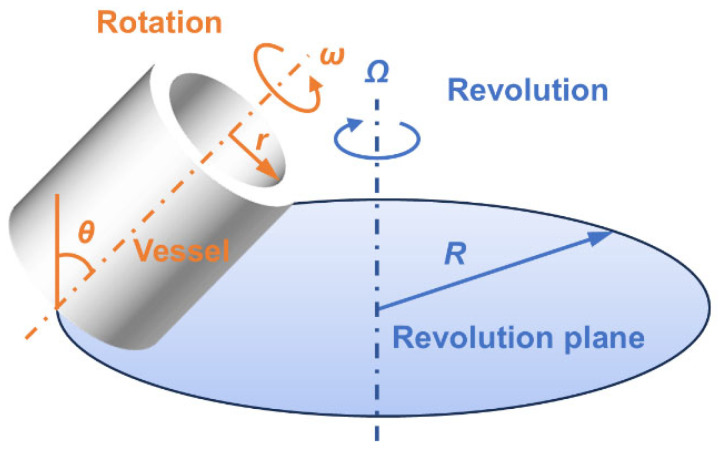
Schematic illustration of the Blade-Free Planetary Mixer (BFPM).

In this study, the BFPM, a schematic illustration of which is shown in Figure 1, was self-developed in the laboratory (The experimental setup is provided in Figure S1 of the Supporting Information). The mixing container was a hollow cylindrical vessel with a capacity of 130 mL, and its axis was inclined at 45° to the horizontal plane. The slurry mass used in each batch was 30 g. The ratio of rotation speed to revolution speed was fixed at 1:1. Under the baseline conditions, the slurry was processed at 1000 rpm for 25 min under vacuum, and the vacuum level was maintained at −100 kPa. The mixer was operated in unidirectional rotation mode. Before formal mixing, a premixing step was performed to ensure sufficient wetting of the solid powders and to avoid powder splashing.

#### 2.2.2. Characterization Methods

Rheological measurements were conducted using an R/S-SST Plus rheometer (Brookfield, Middleboro, MA, USA) equipped with a CP-25 parallel-plate rotor at a gap of 1.0 mm. The flow curves of the slurry were measured in the controlled shear-rate mode over a shear-rate range of 0–50 s^−1^ at 30 °C. Thermal stability was evaluated using a simultaneous TGA/DSC thermal analyzer (Mettler-Toledo, Zurich, Switzerland). The sample mass was 1 mg, the heating rate was 10 K·min^−1^, and the measurements were carried out from 30 to 600 °C under a nitrogen flow rate of 40 mL·min^−1^. Impact sensitivity was measured using a BAM impact sensitivity tester (AIDESAIEN (Beijing) Technology Co., Ltd., Beijing, China) according to GB/T 21567-2008; *Dangerous goods—Test method for impact sensitivity of explosives substance*. Standards Press of China: Beijing, China, 2008. Friction sensitivity was measured using a BAM friction sensitivity tester (AIDESAIEN (Beijing) Technology Co., Ltd., Beijing, China) according to GB/T 21566-2008; *Dangerous goods—Test method for friction sensitivity of explosives substance*. Standards Press of China: Beijing, China, 2008. The slurry temperature was monitored using a K-type thermocouple thermometer (Wenzhou Longwan Zhuangyuan Weibang Hardware Business Department, Wenzhou, China).

Based on the differences in aluminum powder mass fraction among different sampling points, the in-process mixing uniformity was quantitatively evaluated. In this study, the mixing index was adopted as a simple and intuitive engineering indicator to reflect the maximum spatial difference in Al powder mass fraction among the collected samples. Similar simple mixing indices have also been used in previous studies to characterize spatial mixing uniformity in solid–liquid stirred systems [31,32]. The mixing index was defined as follows:

(1) $$M\;=\;\left(\frac{N_{i}}{N_{j}}\right)\times 100\%$$

where *M* is the mixing index of the sample; *N_i_* is the minimum mass fraction of Al powder in the collected samples; and *N_j_* is the maximum mass fraction of Al powder in the collected samples. In addition, to compensate for the limitation of this extreme-value-based index, the relative standard deviation (RSD) was further introduced as a supplementary statistical indicator of sample-to-sample dispersion, defined as follows:

(2) $$RSD\;=\;\frac{SD}{\bar{N}}\times 100\%$$

(3) $$\;SD=\sqrt{\frac{1}{n-1}\sum_{k=1}^{n}{\left(N_{k}-\bar{N}\right)}^{2}}$$

where *RSD* is the relative standard deviation of the mass fraction of Al powder among the collected samples; *SD* is the standard deviation of the mass fraction of Al powder; $\overset{ˉ}{N}$ is the average mass fraction of Al powder in the collected samples; $N_{k}\;$ is the mass fraction of Al powder in the k-th sample; and n  is the total number of collected samples. The mass fraction of Al in the samples was determined using an Agilent 730 inductively coupled plasma optical emission spectrometer (ICP-OES, Agilent, Santa Clara, CA, USA). The emission power was 1 kW, argon was used as the carrier gas, and the plasma gas flow rate was 15 L min^−1^.

## 3. Results and Discussion

### 3.1. Mixing Performance

The morphology of the propellant slurry at different mixing times under the baseline condition of 1000 rpm is shown in Figure S2a (Supporting Information). At the initial mixing stage (0–6 min), solid particles were observed in the central region of the mixing vessel, and obvious solid agglomeration was present. With the further extension of the mixing time, the number of solid particles in the central region gradually decreased. After 10 min of mixing, the residual solid particles gradually disappeared, and the slurry reached a uniform and stable state.

To further evaluate the mixing uniformity of the propellant slurry in the BFPM, slurry samples were randomly collected from 10 different positions at 6, 12, 18, and 24 min during mixing. The mass fraction of aluminum powder in each sample was measured by inductively coupled plasma optical emission spectrometry (ICP-OES). The differences in the mass fraction of aluminum powder across different sampling positions reflect the degree of mixing uniformity. A smaller difference indicates a higher level of uniformity. As the mixing time increased, the mixing index gradually increased, exceeding 90% after 12 min and reaching 95.73% at 24 min, where it gradually leveled off, indicating that the system had become essentially uniform. Correspondingly, the RSD values at 6, 12, 18, and 24 min were 4.47%, 2.70%, 1.91%, and 1.67%, respectively, showing a progressive reduction in sample-to-sample variation and confirming the improved mixing uniformity (Figure S2b, Supporting Information).

### 3.2. Mixing Process Safety

The safety of solid propellants is commonly evaluated by sensitivity to external stimuli, such as heat, impact, and friction [33,34]. Although temperature and apparent viscosity do not directly indicate hazardous outcomes, they reflect energy accumulation, flow behavior, and component distribution in the system, while thermal stability provides additional insight into the potential reaction risk under heating or local heat accumulation conditions [18]. In this section, the safety of the mixing process was assessed from three aspects: the evolution of slurry state, characterized by temperature and apparent viscosity; mechanical sensitivity and thermal stability.

#### 3.2.1. Slurry Property Evolution

**Figure 2:**
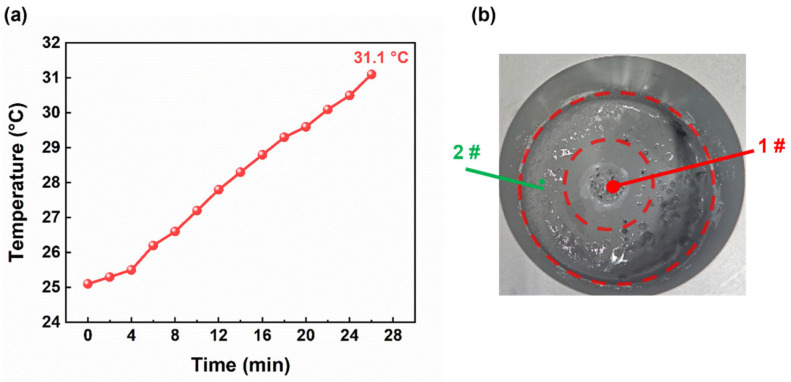
(**a**) Temperature of the propellant slurry. (**b**) Schematic illustration of the sampling positions.

A K-type thermocouple sensor was used to monitor the temperature of the propellant slurry inside the mixing vessel. As mixing proceeded, the temperature increased continuously (Figure 2a). After 26 min, the slurry temperature reached 31.1 °C, which was about 6 °C higher than the initial value. In industrial production, the temperature safety threshold for propellant mixing is generally between 50 °C and 60 °C [35,36]. The final temperature and temperature rise using the BFPM are well below this safety threshold. This indicates that the BFPM produces a limited temperature rise under the present mixing conditions, suggesting a relatively low risk of excessive temperature accumulation during processing.

At the initial mixing stage, the slurry in the vessel was unevenly distributed and showed a clear zonal pattern. It could be roughly divided into a central, solid-particle-rich region and an outer region. To study slurry property evolution during mixing, the apparent viscosity of the slurry in two regions was measured and analyzed at different times under different shear rates. The sampling positions are shown in Figure 2b. Sampling 1# was located at the spiral center of the central region, while sampling 2# was located in the outer region. Positions 1# and 2# were kept fixed at the same horizontal level.

**Figure 3:**
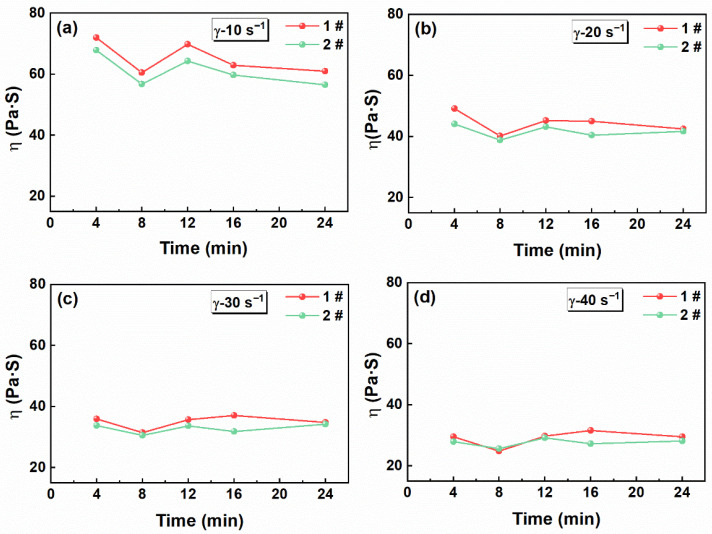
Apparent viscosity of the propellant slurry at different shear rates: (**a**) 10 s^−1^, (**b**) 20 s^−1^, (**c**) 30 s^−1^, (**d**) 40 s^−1^.

Figure 3 shows the evolution of slurry apparent viscosity with mixing time at different shear rates. The results indicated clear regional differences in the apparent viscosity of the slurry. Specifically, the apparent viscosity in the outer region was lower than that in the central region by approximately 5 Pa·s. This difference may be attributed to the combined action of two distinct mechanisms. First, the solid particles are subjected to three main forces during mixing: the centrifugal force generated by rotation, the viscous drag exerted by the binder-rich liquid phase, and the contact friction between particles. Although dense solid particles such as AP and RDX experience larger centrifugal forces relative to the lower-density liquid binder, the mechanical resistance caused by initial particle agglomeration, interparticle friction, and viscous drag slows their outward migration, resulting in the transient enrichment of solid particles in the spiral central region [37]. Simultaneously, the low-viscosity liquid phase migrates more readily toward the vessel wall under centrifugal forces. This solid concentration gradient primarily governs the initially higher viscosity observed in the central region. Second, according to simulation results reported in the literature [23], the flow velocity and local shear rate near the spiral center are relatively low, and at the initial stage of mixing, they are insufficient to overcome particle agglomeration and cohesion. This not only results in a transient enrichment of solid particles in the central region, but also implies that the local shear effect is weak. In contrast, regions near the vessel wall experience higher shear rates [22,38], which promote agglomerate breakup, particle dispersion, and shear thinning. The combined effects of these two mechanisms result in the initially higher viscosity observed in the central region, while the outer region exhibits better dispersive mixing and lower apparent viscosity. Furthermore, this central enrichment phenomenon occurs only during the initial stage of mixing and does not persist as a spatial distribution throughout the mixing process.

As the mixing time increased, the mixer continuously imparted mechanical work and energy to the slurry. Part of this energy promoted particle dispersion, while the rest was consumed in overcoming the resistance at the solid–liquid interface, thus enhancing the wetting of solid particles by the liquid phase [39]. The apparent viscosity gradually decreased. With prolonged mixing, the particle surfaces became more fully wetted, and the centrally agglomerated structure was progressively disrupted under shear. As a result, the internal structure of the slurry gradually became more uniform and stable, and the compositional differences between regions were progressively reduced [22]. However, the viscosity in region 2# remains slightly lower than that in the central region (1#), indicating that differences in local shear rate still exert a certain influence on the slurry viscosity. Regions with higher apparent viscosity generally contained stronger interparticle friction and greater frictional resistance. During mixing, these regions are more prone to local heat accumulation and mechanical propellant stimulation, thereby increasing the process hazard.

#### 3.2.2. Mechanical Sensitivity

**Figure 4:**
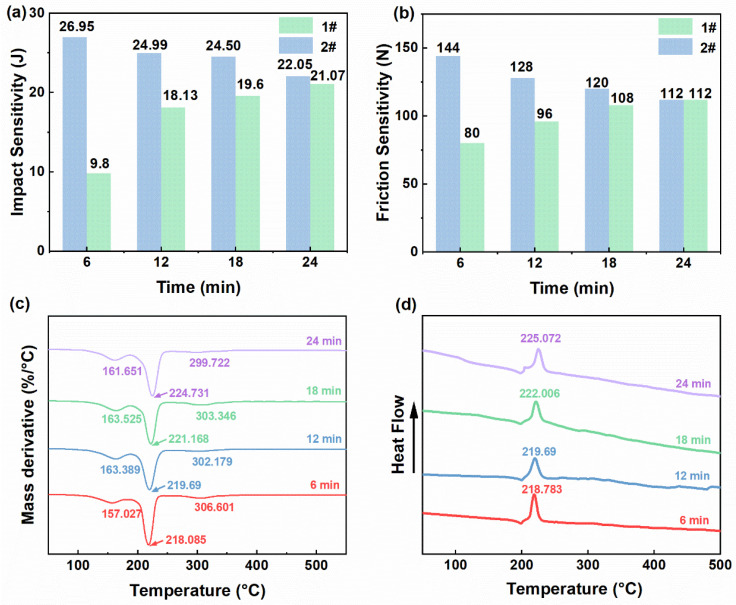
(**a**) Impact sensitivity of the propellant slurry at different mixing times. (**b**) Friction sensitivity of the propellant slurry at different mixing times. (**c**) DTG curves at different mixing times. (**d**) DSC curves at different mixing times.

Figure 4a,b illustrate the mechanical sensitivity results for different regions. The results showed that the impact and friction sensitivities of the slurry changed in a similar manner with mixing time. The mechanical sensitivity in the central region gradually decreased, whereas that in the outer region gradually increased. Specifically, the characteristic value of friction sensitivity in the central region increased from 80 N to 112 N, while that in the outer region decreased from 144 N to 112 N. Similarly, the characteristic value of impact sensitivity in the central region increased from 9.8 J to 21.07 J, whereas that in the outer region decreased from 26.95 J to 22.05 J. Near the end of mixing, the mechanical sensitivity of the slurry became nearly identical in the different regions, suggesting that the regional differences in the slurry were markedly reduced and that the system approached a more uniform state. At the initial mixing stage, agglomeration was present in the vessel’s central region. This region contained a higher proportion of energetic solid components. As mixing proceeded, the solid agglomerates were gradually broken up and continuously migrated and dispersed within the vessel, and the system gradually became more uniform. Because the energetic solid components are the primary sensitive phase for local hot spot formation and reaction initiation under mechanical stimulation, their content directly affects the sample’s impact and friction sensitivities [40]. The mechanical sensitivity, therefore, gradually decreased in the central region and gradually increased in the outer region.

A comparison of the mechanical sensitivity against the apparent viscosity revealed a certain correlation. Regions with higher apparent viscosity in the vessel generally correspond to higher mechanical sensitivity. A plausible interpretation is that high-viscosity regions have greater frictional resistance between solid particles. Under external mechanical stimulation, this may favor localized frictional heating and hot-spot formation, which could increase the sensitivity of the slurry to impact and friction. In addition, high-apparent-viscosity regions are often associated with local enrichment or agglomeration of solid particles, leading to a higher concentration of energetic particles. This further elevates the mechanical sensitivity. Therefore, the rheological properties of the slurry not only influence mixing uniformity, but may also be closely related to local safety-related behavior during the mixing process.

#### 3.2.3. Thermal Stability

The TG results are provided in Figure S3 (Supporting Information). The propellant exhibited a three-stage thermal mass-loss behavior. The first stage, occurring over 126.32–197.83 °C, was mainly associated with the volatilization and initial decomposition of the plasticizer, resulting in a mass loss of approximately 24%. The second stage, spanning 202.70–248.67 °C, was primarily attributed to the decomposition of RDX together with part of the AP, and accounted for a mass loss of approximately 45%. The third stage, occurring over 279.41–361.53 °C, mainly involved the decomposition of AP and PET, with a corresponding mass loss of about 5%. Figure 4c shows the peak temperatures corresponding to each decomposition stage at different mixing times. When the mixing duration was increased, the decomposition peak temperatures of the first and second stages gradually shifted to higher temperatures, and the third stage gradually shifted to lower temperatures. In particular, the thermal decomposition peak temperature of the second stage, which contained a relatively high proportion of energetic components, increased steadily from 218.085 °C to 224.731 °C. One possible explanation is that the energetic crystals became more effectively wetted and coated by the binder and plasticizer as mixing proceeded [40,41,42]. And a higher temperature would be required to initiate thermal decomposition. This interpretation is consistent with the upward shift in the decomposition peak temperature and suggests an improvement in thermal stability.

Meanwhile, the DSC results (Figure 4d) indicate that the exothermic peak temperatures of the propellant slurry at mixing times of 6, 12, 18, and 24 min were 218.783 °C, 219.690 °C, 221.496 °C, and 225.072 °C. The exothermic peak temperature of thermal decomposition increased progressively with mixing time, further confirming that the mixing process had an effect on the propellant’s thermal decomposition behavior. Based on the above results, the safety-related behavior of the propellant slurry during BFPM processing is likely influenced by two key factors: the spatial distribution of energetic solid components and the mixing state between the solid and liquid components.

Under the established basic process conditions, a relatively uniform and safe propellant slurry can be obtained. However, these conditions are only preliminary and have not been systematically optimized in terms of mixing performance and safety. To further enhance the mixing effect and improve process safety, key process parameters such as the mixing speed and rotation mode should be further adjusted and optimized.

### 3.3. Optimization of Mixing Process Conditions

Process conditions such as the mixing speed and rotation mode directly affect the flow behavior and mixing degree of the materials in the equipment, thereby influencing their safety performance. In this section, the process conditions were varied to investigate the effects of different operating conditions on the safety of the mixing process, providing a basis for further optimization.

#### 3.3.1. Rotation Speed

**Figure 5:**
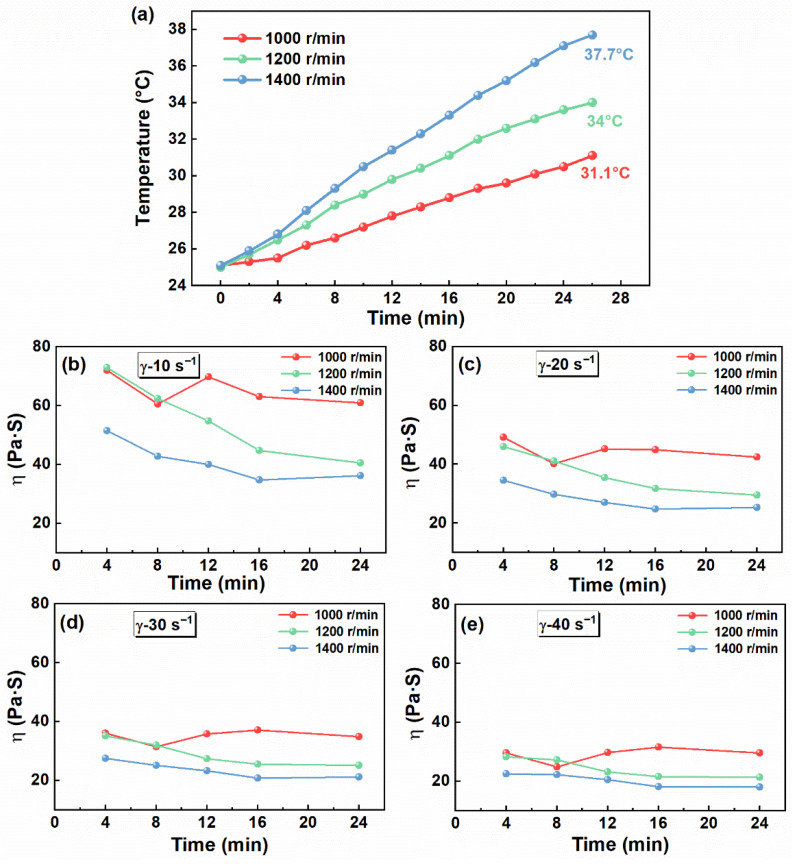
(**a**) Effect of rotation speed on temperature at different mixing times. (**b**–**e**) Effect of rotation speed on apparent viscosity at different mixing times (at shear rates of 10, 20, 30, and 40 s^−1^).

To investigate the influence of rotation speed on mixing process safety, comparative experiments were conducted at three mixing speeds of 1000, 1200, and 1400 rpm. The sampling location was set at position 2#. Figure 5a shows that the propellant slurry temperature rose with increasing rotational speed. In general, a higher rotation speed imparts greater kinetic energy to the powder particles, which can lead to an increase in the mixture temperature [43,44]. After 26 min at a rotational speed of 1400 rpm, the system temperature remained below 40 °C, corresponding to an increase of 12.7 °C compared with the initial temperature. These results indicate that the BFPM produces a limited temperature rise during the mixing process, suggesting favorable thermal controllability under the present conditions.

As shown in Figure 5b–e, the slurry viscosity exhibited different evolution trends at different rotational speeds. At 1000 rpm, the viscosity first increased and then decreased, suggesting local solid particle enrichment at the initial stage. In contrast, at 1200 and 1400 rpm, once mixing began, the viscosity decreased continuously. The slurry viscosities at 1000 and 1200 rpm were similar at the beginning of mixing; however, after 10 min, that at 1200 rpm became clearly lower. At 1400 rpm, the viscosity was significantly lower than that under the other two conditions at the initial stage. Overall, increasing rotation speed enhanced shear and dispersion during mixing and may have promoted system transition toward chaotic mixing [22]. This accelerated homogenization and caused the slurry viscosity to decrease more rapidly, thereby avoiding excessively high local viscosity.

**Figure 6:**
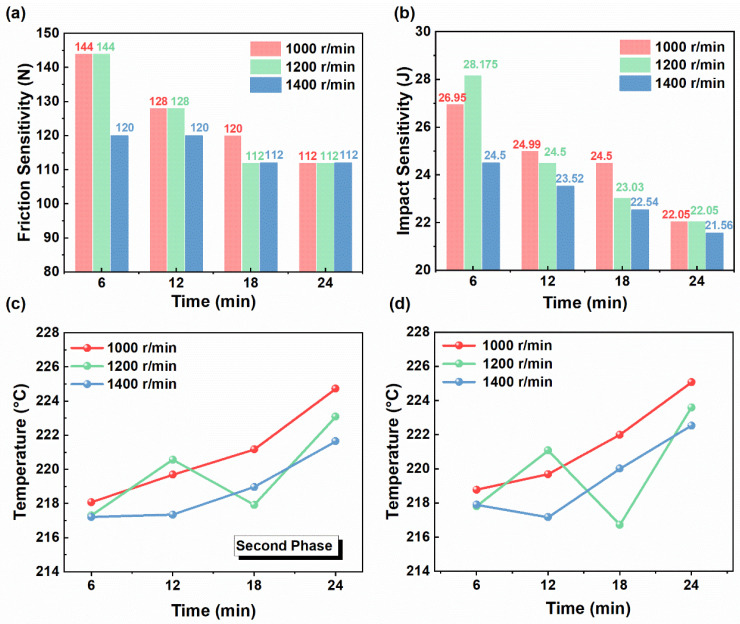
(**a**) Effect of rotation speed on impact sensitivity at different mixing times. (**b**) Effect of rotation speed on friction sensitivity at different mixing times. (**c**) Effect of rotation speed on DTG decomposition peak temperature at different mixing times. (**d**) Effect of rotation speed on DSC exothermic peak temperature curves at different mixing times.

Figure 6a,b show the mechanical sensitivity results at the 2# region. For friction sensitivity, the characteristic values at 1000 and 1200 rpm both decreased from 144 N to 112 N, whereas that at 1400 rpm changed from 120 N to 112 N. A similar trend was observed for impact sensitivity. The characteristic value decreased from 26.95 J to 22.05 J at 1000 rpm, from 28.175 J to 22.05 J at 1200 rpm, and from 24.5 J to 21.56 J at 1400 rpm. These results show that the mechanical sensitivity in region 2# exhibited different evolution trends under different rotation speeds. At 1200 rpm, the mechanical sensitivity increased relatively rapidly during mixing. By contrast, at 1400 rpm, it was already at a relatively high level at the initial stage. As mixing proceeded, the differences in mechanical sensitivity among the three rotation-speed conditions gradually diminished. This behavior may be related to faster material redistribution within the vessel at higher rotational speed, so that the local composition in region 2# changed more rapidly during mixing. The local content of energetic solid components in this region may have increased more quickly at the early stage, which could explain why the sensitivity at 2# reached a relatively high level sooner under higher rotational speed. As mixing proceeded, the differences in mechanical sensitivity among the three rotational-speed conditions gradually decreased, suggesting that the slurry became progressively more uniform.

Thermal analysis of the propellant slurry in region 2# was performed, and the changes in the second-stage DTG and DSC decomposition peak temperatures are shown in Figure 6c,d. At 1000 rpm, the second-stage decomposition peak temperature in the DTG curve increased continuously from approximately 218.085 °C to 224.731 °C, showing the most pronounced upward trend. This indicates that, under this condition, the thermal stability of the system improved steadily with mixing time. Although the peak temperatures at 1200 and 1400 rpm also showed an overall increasing trend, noticeable fluctuations occurred during the process, and the final peak temperatures were respectively 223.094 °C and 221.652 °C, both lower than that at 1000 rpm. The DSC results followed a similar trend to those of the DTG. At the end of mixing, the highest DSC decomposition peak temperature was also observed at 1000 rpm, reaching 225.072 °C.

These results suggest that, at 1000 rpm, the dispersion, wetting, and coating of solid particles proceeded more steadily. The thermal stability of the system increased more continuously. Although increasing the rotational speed is beneficial for intensifying mixing and reducing excessively high local viscosity, an excessively high speed may also alter the interfacial contact state among the components. Under such conditions, enhanced particle collision, friction, and heat generation may promote particle adhesion or the formation of local compact masses [22], which could adversely affect the coating state of energetic particles and make thermal decomposition easier to initiate. Consequently, the decomposition peak temperature shifts to a lower value. Therefore, increasing the mixing speed is beneficial for improving mixing efficiency, but an excessively high speed may adversely affect the thermal stability of the system.

#### 3.3.2. Rotation Mode

**Figure 7:**
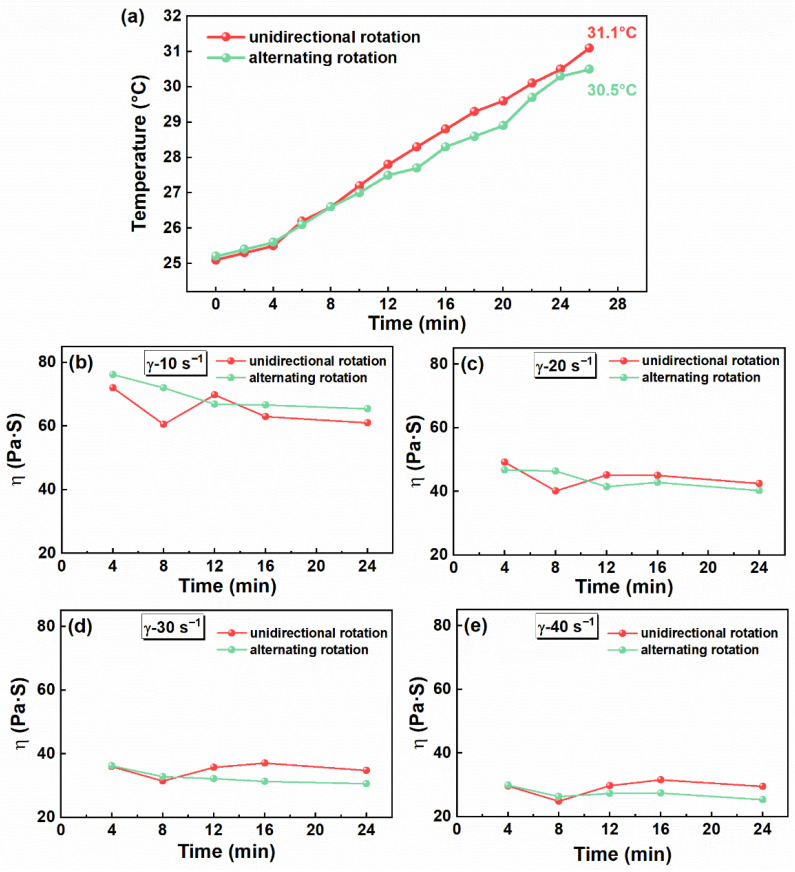
(**a**) Effect of rotation mode on temperature at different mixing times. (**b**–**e**) Effect of rotation mode on apparent viscosity at different mixing times (at shear rates of 10, 20, 30, and 40 s^−1^).

The BFPM can operate in two rotation modes: unidirectional and alternating rotation. In the alternating-rotation mode, the rotation direction was reversed every 1 min throughout the mixing process. To evaluate the influence of rotation mode on propellant mixing process safety, the temperature, viscosity, mechanical sensitivity, and thermal stability of the slurry were examined under both conditions. The research method and sampling region were consistent with those used in the previous study on rotation speed. Figure 7a shows that the system temperature under alternating rotation was consistently lower than that under unidirectional rotation. After 24 min of mixing, the corresponding temperatures were 30.5 °C and 31.1 °C. Under unidirectional rotation, the propellant slurry tends to develop a relatively stable flow structure under the combined action of centrifugal force and viscous drag. In this case, near-wall high-shear regions can be maintained for a longer time, so that mechanical energy is more continuously converted into heat through viscous dissipation, leading to a gradual increase in system temperature. By contrast, under alternating rotation, periodic reversal repeatedly interrupts the established flow structure and forces the slurry to undergo deceleration, reorientation, and re-acceleration. Although this process also requires mechanical work, it weakens the persistence of stable high-shear regions. Relevant studies have reported that, for highly concentrated dense suspensions, oscillatory shear or reversal of the shear direction can lead to decreases in viscosity and viscous dissipation, and this reduction has been attributed to changes in particle contact networks and microstructure under unsteady shear [45,46,47]. Based on the above analysis, it is reasonable to infer that the interruption of sustained high-shear deformation under alternating rotation may reduce local heat accumulation, which could explain the slightly smaller temperature rise observed in the system.

Figure 7b–e further illustrate the differences in mixing behavior between the two operating conditions. Under alternating rotation, the apparent viscosity decreased continuously and became significantly lower than that under continuous unidirectional rotation after more than 10 min of mixing. This behavior may be related to the repeated reversal of the rotation direction, which likely disturbed the established flow structure and promoted material exchange within the slurry. Such unsteady flow may also have facilitated particle dispersion and accelerated the breakdown of locally viscous regions, thereby contributing to a faster decrease in apparent viscosity. Therefore, alternating rotation not only effectively suppresses temperature rise, but also improves mixing performance and reduces local frictional resistance.

**Figure 8:**
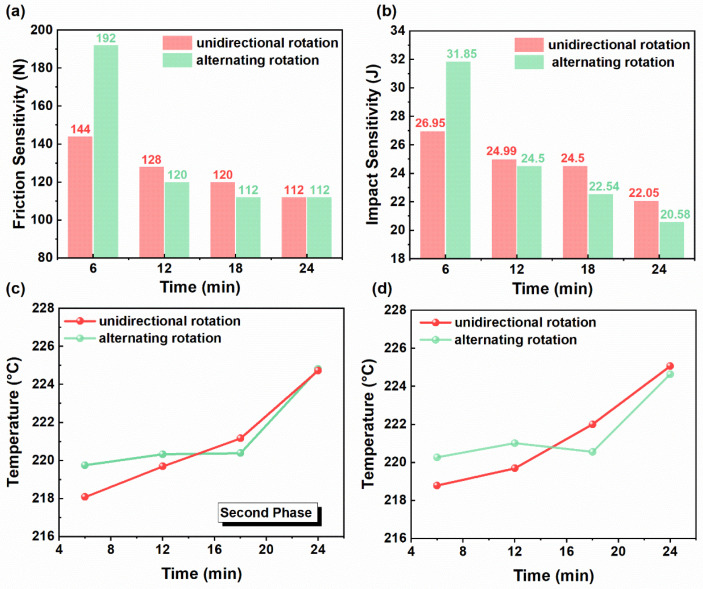
(**a**) Effect of rotation mode on impact sensitivity at different mixing times. (**b**) Effect of rotation mode on friction sensitivity at different mixing times. (**c**) Effect of rotation mode on DTG decomposition peak temperature at different mixing times. (**d**) Effect of rotation mode on DSC exothermic peak temperature curves at different mixing times.

The mechanical sensitivity results (Figure 8a,b) for the slurry sampled from the 2# region showed a faster evolution under alternating rotation. At the beginning of mixing, the characteristic values under alternating rotation were higher than those under unidirectional rotation (192 N vs. 144 N for friction sensitivity, 31.85 J vs. 26.95 J for impact sensitivity), indicating lower local sensitivity initially. This initial difference may be attributed to incomplete mixing at the early stage, with a high degree of deviation in the distribution of energetic solid components. As mixing proceeded, the characteristic values under alternating rotation decreased more rapidly than those under unidirectional rotation. Specifically, the friction sensitivity decreased from 192 N to 112 N, and the impact sensitivity decreased from 31.85 J to 20.58 J under alternating rotation. In comparison, under unidirectional rotation, the friction sensitivity gradually decreased from 144 N to 112 N, and the impact sensitivity decreased from 26.95 J to 22.05 J. Moreover, after 6 min of mixing, the characteristic values under alternating rotation became lower than those under unidirectional rotation at the same time, reflecting a faster increase in local sensitivity.

The thermal analysis results (Figure 8c,d) show that at the initial stage of mixing, the second-stage decomposition peak temperatures in the DTG and DSC curves were relatively higher under alternating rotation, indicating higher thermal stability of the system. As the mixing time increased, although the thermal stability under alternating rotation exhibited a brief downward trend, it subsequently increased rapidly. Overall, alternating rotation tended to provide higher thermal stability for the propellant slurry than unidirectional rotation. The periodic reversal induced by alternating rotation accelerates material dispersion within the system and promotes a more rapid spatial redistribution of energetic solid components. Consequently, the energetic solid content at position 2# increases more quickly, resulting in a faster increase in mechanical sensitivity. Meanwhile, the continuous disturbance of the flow field also facilitates the rapid wetting and coating of energetic crystals by the binder and plasticizer, thereby enhancing thermal stability. Overall, both increasing the rotation speed and adopting alternating rotation promote the dispersion of energetic solid particles, reduce local apparent viscosity, and improve the mechanical sensitivity distribution. However, excessively high rotation speed may lead to a decline in slurry thermal stability.

In selecting the practical optimal processing condition, safety should not be considered as the sole objective; mixing performance should also be taken into account. Compared with 1000 rpm, 1200 rpm promoted particle dispersion and homogenization more effectively, reduced local apparent viscosity more rapidly, and improved the overall mixing state of the slurry. Compared with 1400 rpm, it avoided a more pronounced loss of thermal stability and excessive thermal accumulation. In addition, alternating rotation further enhanced material redistribution, accelerated the reduction in local viscosity, and slightly suppressed the temperature rise. Although the impact sensitivity measured at region 2# under alternating rotation was somewhat higher at the final stage, this result only reflects the local evolution of that sampling region and mainly indicates a faster redistribution of energetic solid components, rather than poorer overall process safety of the whole slurry. Meanwhile, considering the overall evolution of thermal stability, the slurry under alternating rotation showed a more favorable trend. Therefore, 1200 rpm combined with alternating rotation was regarded as a balanced and effective processing condition, providing a favorable compromise between mixing efficiency, process controllability, and acceptable safety.

## 4. Conclusions

This study systematically investigated the mixing performance and process safety of composite solid propellant slurry in a Blade-Free Planetary Mixer (BFPM). The results showed that the BFPM could achieve safe, efficient, and uniform mixing of the slurry. Under the baseline condition, the mixing index reached 95.73% after 24 min, indicating that the system had become essentially uniform. In addition, the temperature rise during mixing remained low, demonstrating good process safety. The safety of the slurry during mixing was mainly governed by two factors: the spatial distribution of energetic solid components and the mixing state of the solid and liquid phases. Mechanical sensitivity showed a certain correlation with apparent viscosity, and regions with higher apparent viscosity generally exhibited higher mechanical sensitivity. By contrast, thermal stability mainly depended on the dispersion, wetting, and coating state of solid particles in the liquid phase. Further process optimization showed that increasing the rotational speed and adopting alternating rotation accelerated particle dispersion and reduced local apparent viscosity. However, excessively high rotational speed adversely affected thermal stability. Considering both mixing performance and process safety, 1200 rpm combined with alternating rotation was identified as the most suitable operating condition. These results provide experimental support for the application of BFPM in the safe mixing of composite solid propellants and offer useful guidance for process optimization and safety control. However, the present study was limited to one representative formulation and laboratory-scale operating conditions. Further work is still needed to examine a wider formulation range. Scale-up experiments are also required to validate the proposed conclusions under larger-scale processing conditions.

## Data Availability

The original contributions presented in this study are included in the article/Supplementary Materials. Further inquiries can be directed to the corresponding author.

## Supplementary Materials

The following supporting information can be downloaded at https://www.mdpi.com/article/10.3390/ma19081672/s1. Figure S1. (a) Schematic illustration of the Blade-Free Planetary Mixer (BFPM) experimental setup. (b) Mixing vessel. Figure S2. (a) The morphology of the propellant slurry at different mixing times under the rotation speed of 1000 rpm. (b) Mixing index–time curve and relative standard deviation–time curve. Figure S3. TG curves of the propellant slurry during the mixing process.
