# Supplementary material for: Mixing Safety of Composite Solid Propellant Slurry in a Blade-Free Planetary Mixer
Source: Materials (Basel). 2026 Apr 21;19(8):1672. doi: 10.3390/ma19081672 (PMC13117734; doi:10.3390/ma19081672)
Supplement: Supplementary file 1 [file materials-19-01672-s001.zip › materials-4244283-supplementary.pdf]

Supplementary Materials For "*Mixing Safety of Composite Solid Propellant Slurry in a Blade-Free Planetary Mixer*"

Yuncheng Li [1], Qingjun Wang [1], Hanyu Chen [1], Yuanwei Xi [2], WeiBin Tao [2], Dayong Li [3] and Min Xia *[1,4]

[1] School of Materials Science and Engineering, Beijing Institute of Technology, Beijing 100081, China;
3220231524@ bit.edu.cn (Y.L.)
[2] Liaoning Qing Yang Chemical Industry Corporation, Liaoyang 111002, China;
[3] China North Chemical Research Academy Group Co., Ltd, Beijing 100089, China;
[4] Key Laboratory of High Energy Density Materials, Ministry of Education, Beijing Institute of Technology, Beijing 100081, China
* Correspondence: xminbit@bit.edu.cn

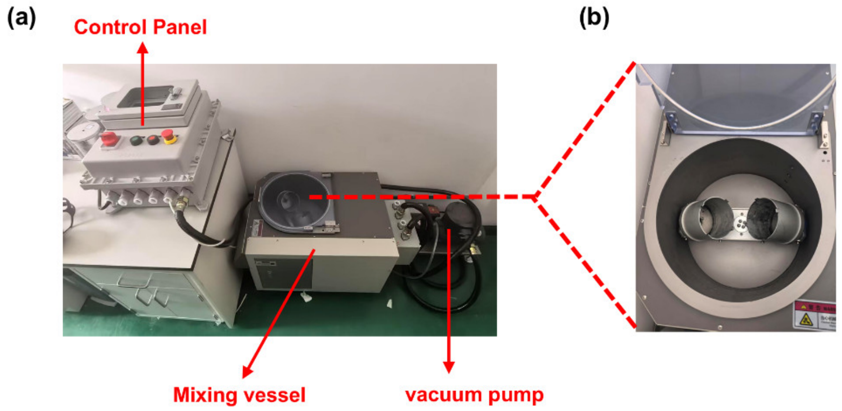

Figure S1. (a) Schematic illustration of the Blade-Free Planetary Mixer (BFPM) experimental setup. (b) Mixing vessel.

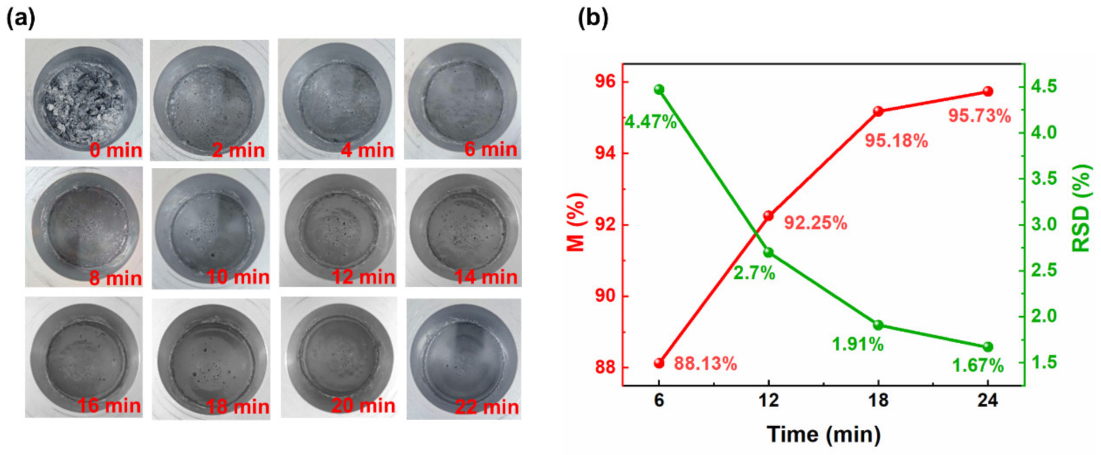

Figure S2. (a) The morphology of the propellant slurry at different mixing times under the rotation speed of 1000 rpm. (b) Mixing index–time curve and relative standard deviation–time curve

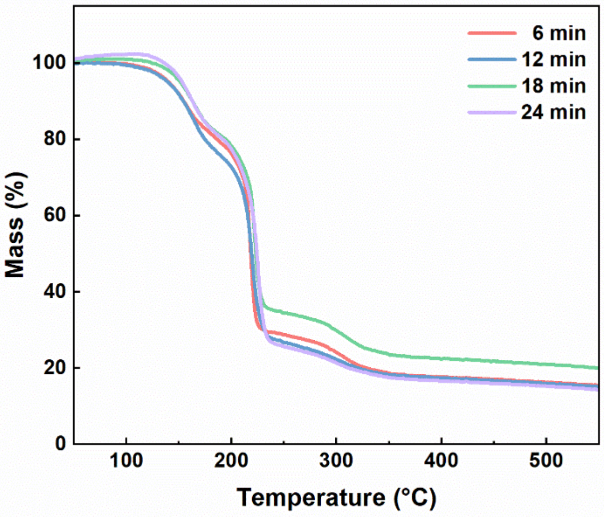

Figure S3. TG curves of the propellant slurry during the mixing process.
